# On the role of generative artificial intelligence in the development of brain-computer interfaces

**DOI:** 10.1186/s42490-024-00080-2

**Published:** 2024-05-02

**Authors:** Seif Eldawlatly

**Affiliations:** 1 Computer and Systems Engineering Department, Faculty of Engineering, Ain Shams University, 1 El-Sarayat St., Abbassia, Cairo, Egypt; 2 Computer Science and Engineering Department, The American University in Cairo, Cairo, Egypt

**Keywords:** Brain-computer interface, Generative artificial intelligence, Variational autoencoders, Generative adversarial networks, Transformers, Diffusion model

## Abstract

Since their inception more than 50 years ago, Brain-Computer Interfaces (BCIs) have held promise to compensate for functions lost by people with disabilities through allowing direct communication between the brain and external devices. While research throughout the past decades has demonstrated the feasibility of BCI to act as a successful assistive technology, the widespread use of BCI outside the lab is still beyond reach. This can be attributed to a number of challenges that need to be addressed for BCI to be of practical use including limited data availability, limited temporal and spatial resolutions of brain signals recorded non-invasively and inter-subject variability. In addition, for a very long time, BCI development has been mainly confined to specific simple brain patterns, while developing other BCI applications relying on complex brain patterns has been proven infeasible. Generative Artificial Intelligence (GAI) has recently emerged as an artificial intelligence domain in which trained models can be used to generate new data with properties resembling that of available data. Given the enhancements observed in other domains that possess similar challenges to BCI development, GAI has been recently employed in a multitude of BCI development applications to generate synthetic brain activity; thereby, augmenting the recorded brain activity. Here, a brief review of the recent adoption of GAI techniques to overcome the aforementioned BCI challenges is provided demonstrating the enhancements achieved using GAI techniques in augmenting limited EEG data, enhancing the spatiotemporal resolution of recorded EEG data, enhancing cross-subject performance of BCI systems and implementing end-to-end BCI applications. GAI could represent the means by which BCI would be transformed into a prevalent assistive technology, thereby improving the quality of life of people with disabilities, and helping in adopting BCI as an emerging human-computer interaction technology for general use.

## Background

A Brain-Computer Interface (BCI) is a system that allows direct communication between the brain and the external environment [1]. BCI is a promising emerging technology that could help persons with disabilities regain, at least partially, their lost abilities. It could be of help when the disability is not caused by a brain damage. BCIs can be classified into invasive and non-invasive. Invasive BCIs have demonstrated success in a multitude of tasks given the high spatiotemporal resolution of acquired brain activity using these systems, which could discern the response of single neurons in the brain [2]. However, this is associated with the high-risk of performing a surgery that aims at implanting electrodes in the brain, which could be required for some medical conditions. On the other hand, non-invasive BCIs that mainly rely on recording Electroencephalogram (EEG) signals by wearing a head cap of electrodes on the scalp represent a safer and a more practical solution [3]. Recent advances in BCIs have demonstrated the efficacy of translating recorded EEG signals into actions that represent users’ intentions. Successful examples of BCIs include EEG-speller systems [4–7], wheelchair control [8, 9], upper- and lower-limb prosthetics control [10–12], robot control [13, 14], and brain-controlled games [15]. In addition, BCI has also been demonstrated to represent a novel human-computer interaction technology that is not limited only to people with disabilities [16–18].

Despite the success BCI has demonstrated in tackling many challenges faced by people with disabilities, the adoption of such a solution as a long-term assistive technology has been lacking [19]. This can be attributed to a number of challenges that hinder the widespread use of BCI. First, calibrating BCIs requires recording an extensive amount of EEG data from users. This is needed to train the machine learning algorithms that are typically used in such a context to recognize the user’s intentions. However, recording EEG calibration data for long periods of time is inconvenient for users, especially for patients with medical or mental conditions. Second, EEG systems that provide high channel count with high resolution are typically used in hospitals for clinical purposes and of high cost. These systems are not suitable for everyday use that requires the portability of the EEG recording system. Recently, a number of EEG headsets have been made commercially available at reasonable prices [20]. These headsets overcome the aforementioned limitation by providing portable wireless units with sensors that do not require the application of a conductive gel. However, they mainly offer a limited number of recording channels at low spatiotemporal resolution. Finally, developing universal BCI systems calibrated using data of subjects other than the user has proven to be challenging due to the inter-subject variability that is typically observed in BCI tasks [21]. This requires recording EEG signals from each subject to calibrate the BCI system and operate it in a subject-independent manner.

Since the inception of BCI, multiple efforts in the community have been directed towards tackling the aforementioned challenges [22]. Artificial intelligence (AI) techniques, and machine learning in particular, have represented a core component in any BCI system to help in recognizing the user’s target command. Recently, advances in the Generative AI (GAI) field have demonstrated significant success in a multitude of long-standing tasks that is revolutionizing many aspects of today’s life [23]. GAI has been used in generating different types of realistic data including text, images, audio and video [24]. GAI techniques rely on deep learning models with an extremely large number of tunable parameters that are trained using large amounts of data.

In this article, the recent adoption of GAI techniques in application to non-invasive BCIs is highlighted. First, an overview is provided of how GAI techniques are employed to generate synthetic data to augment the limited amount of EEG data typically recorded in BCI applications. Such augmentation enhances the performance of machine learning algorithms typically used in different BCI applications. Second, a description is provided of how GAI techniques are used to generate EEG signals with high spatiotemporal resolution from EEG signals recorded with lower spatiotemporal resolution. Third, examples are given for using GAI techniques to enhance the cross-subject performance by generating subject-invariant features or signals. Finally, using GAI techniques in developing end-to-end applications is demonstrated, were GAI techniques are used to transform the recorded EEG signals to other forms including audio and images.

There are multiple GAI models that have been utilized in the aforementioned four applications of GAI in BCI development; however, of particular interest to this article are Variational Autoencoders (VAEs), Generative Adversarial Networks (GANs), transformers and diffusion models [24, 25]. A VAE consists of two back-to-back networks: An encoder network which encodes the input data into a latent space of lower dimensions by mapping the input data into a latent space distribution, followed by a decoder network that maps the latent space back to the input data space [26]. Once trained in a regularized manner, the decoder can be used independently to generate new data. A GAN, in its most basic form, consists of two networks: a generator network followed by a discriminator network [27]. The generator is trained to synthesize outputs that are as similar as possible to the input data. On the other hand, the discriminator is trained to discriminate between the input real data and the synthetic (fake) data generated by the generator. The inability of the discriminator to differentiate between real and synthesized data indicates the success of the training of the generator. Once the training converges, the generator can be independently used to generate additional data similar in nature to the input data. A transformer, similar to VAEs, uses the concept of an encoder followed by a decoder. However, in addition to using such a general architecture, an attention mechanism is employed in which the decoder would be able to focus on relevant components of the input data sequence [28]. Transformers are by design developed to handle sequential data. Finally, diffusion models attempt in one forward direction (resembling encoders) to add Gaussian noise to the input data along a Markov chain, while in the backward direction (resembling decoders) attempt using a neural network to reverse the process of noise addition to generate a data point from the same space of the input data [29].

This article provides an overview of the use of GAI techniques to enhance BCI systems. Using GAI in developing BCI systems represents one step forward towards the widespread adoption of BCI as a reliable assistive/ human-computer interaction technology.

### GAI for data augmentation

**Fig. 1:**
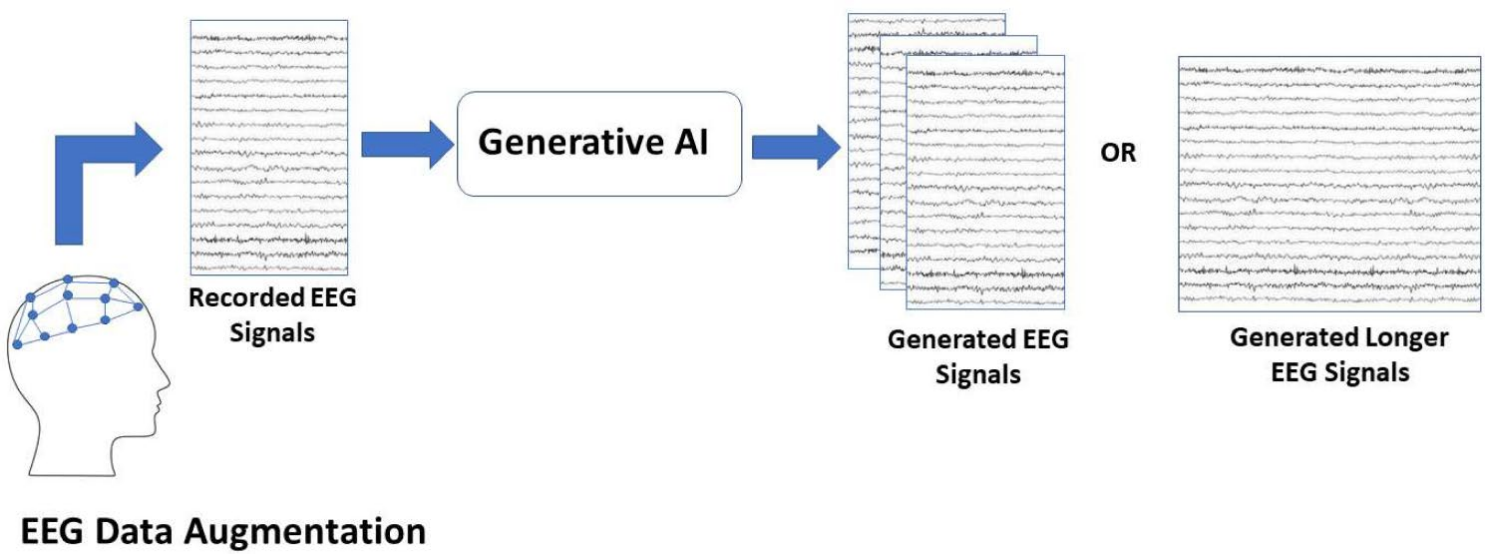
Using GAI for EEG data augmentation

GAI techniques have been demonstrated to augment limited EEG data recorded for BCI systems calibration by generating synthetic EEG data as illustrated in Fig. 1. BCI systems rely on different EEG patterns in their operation. One EEG pattern that has been utilized in multiple applications is the P300 pattern [30]. This is a positive peak that appears in EEG signals approximately 300ms after the presentation of a rare anticipated stimulus. Such a stimulus could be auditory or visual. P300-based BCI systems typically rely on a machine learning binary classifier to discriminate between the P300 pattern and background activity (non-P300) [4]. To achieve acceptable subject-dependent classification accuracy, a large number of trials is required from the subject where one epoch typically consists of more than 10 trials for a single target. As a result, GAI techniques were introduced to augment the limited amount of data. Different GAN architectures have been utilized in this task including Deep Convolutional GAN (DCGAN), Conditional GAN (cGAN), Auxiliary Classifier GAN (ACGAN) and Gradient Penalty-based Wasserstein GANs (WGAN-GP) [31–34]. These GANs rely on Convolutional Neural Network.

(CNN) architecture for both the Generator and the Discriminator, and/or Recurrent Neural Networks (RNNs). Synthetic data generated using GANs when added to the real recorded data led to an increase in the P300 recognition accuracy by 10–18% when either balancing the two classes of P300 and non-P300 or generating synthetic data that is at least 4 times the size of the real recorded data. In addition, reducing the training data size, in terms of the number of targets, by 30% and compensating for the dropped data using GANs does not significantly impact the accuracy. These results indicate that using GAI can reduce the amount of training data needed, leading to a shorter calibration time and less inconvenience for the users.

Another EEG pattern that has been utilized in a multitude of BCI applications is the Steady-state Visual Evoked Potential (SSVEP). This pattern is characterized by recording EEG signals with a frequency spectrum that resembles the frequency spectrum of a presented flickering visual stimulus [35]. In many SSVEP-based BCIs, machine learning classifiers are used to discriminate between the spectral signature of the SSVEPs of different flickering frequencies. One use of GAI in this context is, similar to that used with P300 patterns, to generate synthetic data to increase the size of the training dataset of the classifiers. Multiple GAI models were used in this task including VAE, DCGAN, WGAN and StarGAN to generate synthetic data in the time-domain as well as in the frequency-domain improving the SSVEP recognition accuracy by 2–20% [36–38]. Another use of GAI with SSVEP patterns is to generate synthetic data to increase the duration of the recorded real data which can help in obtaining a better spectrum estimation as well as reduce the amount of data to be recorded from the users for system calibration [39]. A Long Short-term Memory (LSTM)-based GAN is used along with ACGAN to generate signals of longer duration, which increases the SSVEP recognition accuracy by $\sim$ 30%.

Motor Imagery (MI) represents one of the main BCI paradigms that relies on recognizing spectral patterns in the EEG that correspond to imagined movements [40]. Event Related Synchronization (ERS) and Event Related Desynchronization (ERD) patterns are recognized mainly in signals recorded from electrodes placed around the motor cortex brain area in both hemispheres. The majority of the literature on using GAI in BCI data augmentation is on augmenting MI data. Multiple studies considered using different GAI techniques in augmenting the time-domain EEG signals using VAE, DCGAN, and conditional WGAN (cWGAN) leading to an enhancement in the MI pattern classification accuracy of 1–18% depending on the dataset, the GAN architecture and the classification model [41–43]. Another approach is to first transform the recorded EEG signals into the frequency domain using Short-time Fourier Transform (STFT) and subsequently represent each trial as an image of the spectrum versus time. GAI is then used to generate synthetic images of STFTs to augment the recorded data. Using this approach, vanilla GAN and DCGAN were examined in this task reaching an enhancement in the overall MI pattern classification accuracy of 2.5–12% [44, 45].

Finally, it is worth mentioning that GAI has been used for BCI data augmentation in other tasks. For instance, to augment EEG data in the task of emotion recognition, the OpenAI Improved-diffusion model has been examined [46]. Additionally, multiple GAN approaches were examined in the same task including cWGAN and ACGAN increasing emotion recognition accuracy by 1.5–20% [47, 48].

### GAI for EEG resolution enhancement

**Fig. 2:**
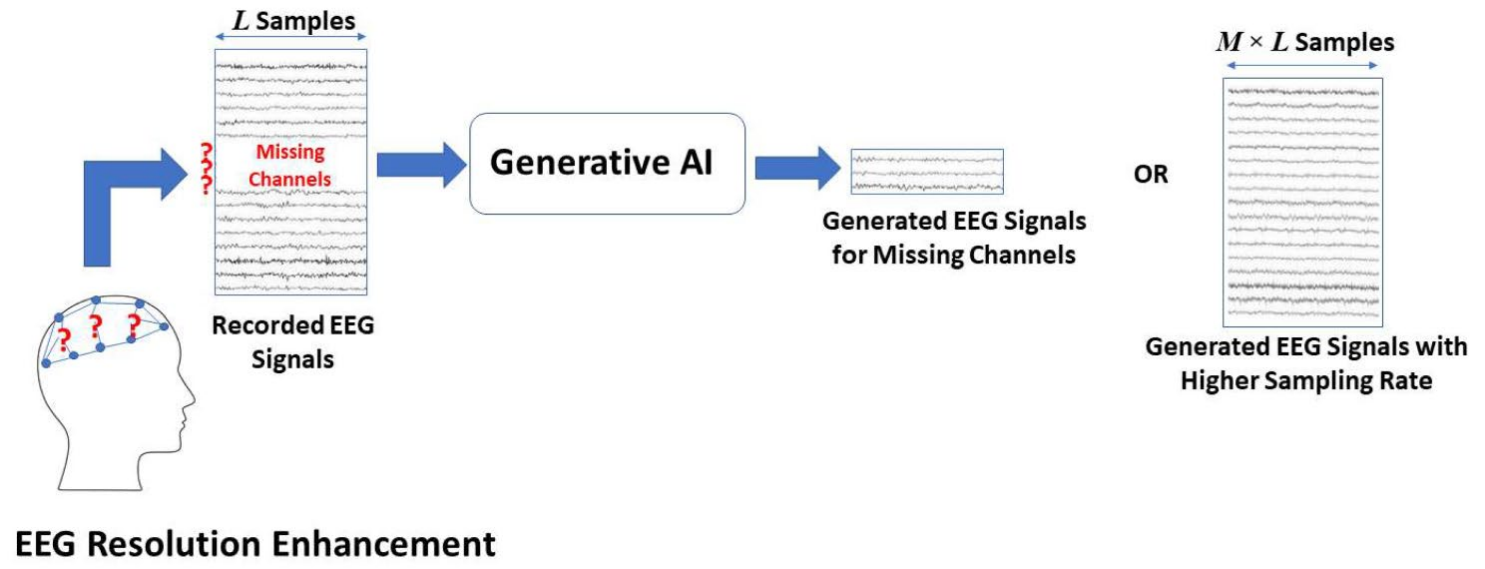
Using GAI for enhancing EEG spatial and temporal resolutions

One of the applications in which GAI has demonstrated significant improvement in domains such as computer vision is generating super-resolution images from lower resolution versions [49]. In EEG signals analysis for BCIs, GAI has been applied to enhance both the spatial and temporal resolutions of the recorded EEG signals as illustrated in Fig. 2. First, considering the spatial resolution enhancement, recording EEG signals with high spatial resolution in terms of the number of electrodes used in the headset and the proximity of the electrodes is typically challenging and requires expensive equipment that might not be suitable for practical daily-usage BCI applications [50]. As a result, a number of studies has explored using GAI to enhance the spatial resolution of EEG signals. This is typically done by estimating the signals from electrodes that are not present in the EEG recording headset. For instance, multiple studies have employed Deep CNNs in this task in which the input is the data recorded from a limited number of electrodes and the target.

output of the CNN is signals from a larger number of electrodes [51–53]. In these studies, the number of input electrodes was as low as 4 electrodes only while the number of output synthesized electrodes reached 60 electrodes. In addition, WGAN was also explored in the same task where signals from only 25% of the channels were used to generate signals from the remaining 75%. Surprisingly, this results in a minimal reduction of only 9% in the accuracy of mental imagery classification when comparing the performance achieved using the generated data to that achieved using the ground-truth data [54]. The generated high spatial-resolution signals demonstrated strong similarity with the reference signals indicating the efficacy of using GAI in this challenging task.

Another perspective of enhancing EEG spatial resolution is to map EEG signals to the underlying neural activity that could be recorded using other invasive neural data recording techniques. EEG is considered a low-pass filtered and distorted version of single-neuron activity [55]. Using GAI to map EEG to other underlying neural signals not only could help enhancing the spatial resolution of EEG for better BCI performance, it could also help in understanding how single neuron activity and local field potentials brain dynamics map to EEG. Deep neural networks comprised of residual networks (ResNet) and long short-term memory (LSTM) were utilized in this task to map EEG to mesoscale neural activity, achieving a mean correlation of 0.83 comparing the generated signals to the ground-truth [56]. Other studies explored using autoencoders, vanilla GANs, and conditional GANs to map EEG to invasively recorded intracranial EEG [57, 58]. Results reported by Abdi-Sargezeh et al. demonstrated the utility of using GANs to detect interictal epileptiform discharges with an accuracy reaching 76%, outperforming previous approaches [59].

In addition to enhancing EEG spatial resolution, GAI was also employed in enhancing the temporal resolution of the recorded EEG. This could be used to generate high temporal resolution EEG signals from lower temporal resolution ones. This could alleviate the challenges associated with developing high temporal resolution EEG recording devices such as the complexity and high cost of developing and operating such devices [60]. Vanilla GAN and WGANs were used to double the sampling rate of the recorded EEG leading to an enhancement in the classification accuracy of a MI dataset by$\sim$4% [43, 61]. Although not being thoroughly examined as the enhancement of spatial resolution, enhancing EEG temporal resolution using GAI techniques is promising. Further advances in this area could help in reducing the cost of EEG recording devices by augmenting the capabilities of low temporal-resolution devices.

### GAI for cross-subject performance enhancement

**Fig. 3:**
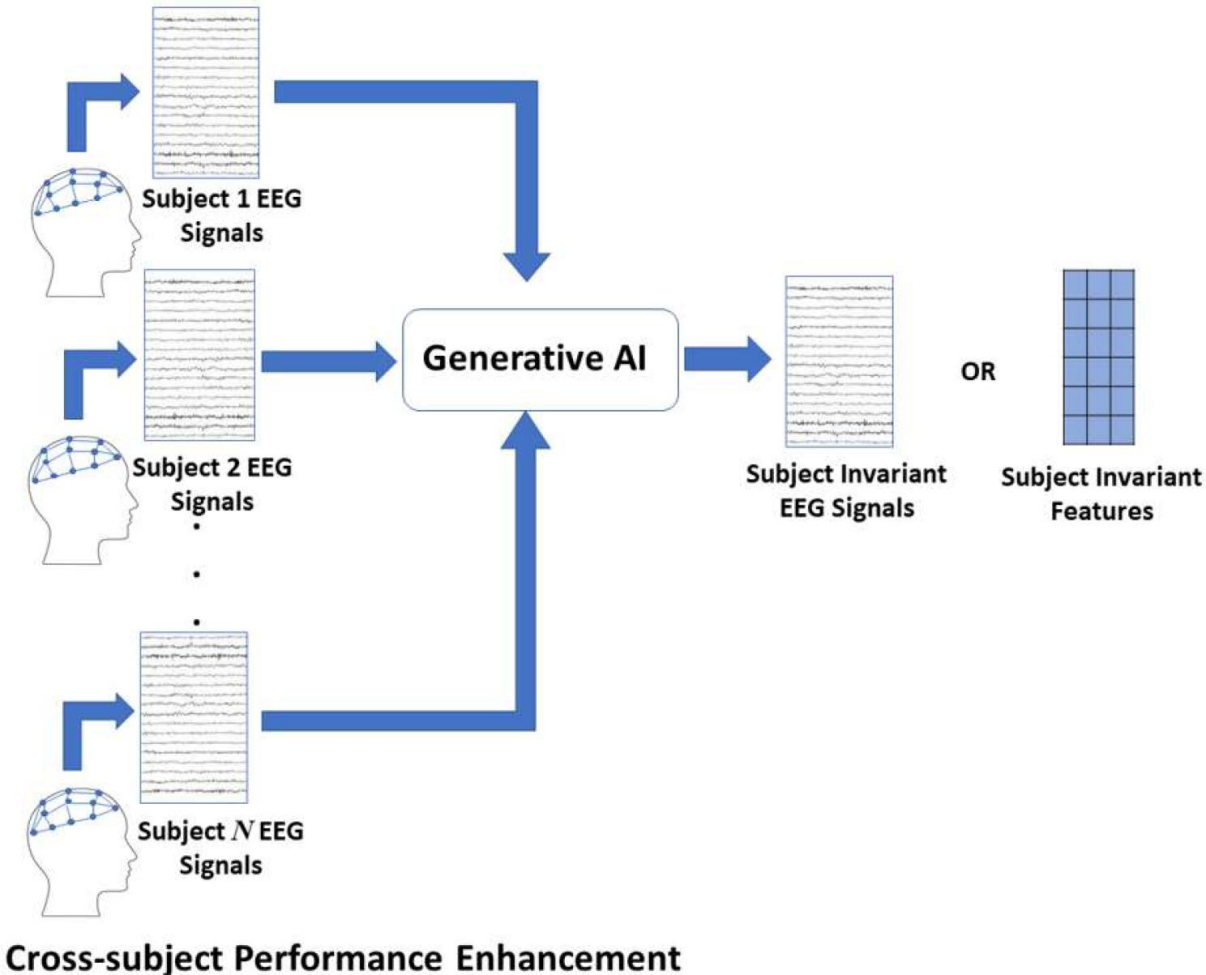
Using GAI to enhance BCI cross-subject performance

Inter-subject variability represents one of the main challenges hindering the widespread use of BCI. Such significant variability requires recording a significant amount of training (calibration) data from each BCI user to train a subject-dependent model to be used to determine the user’s command or intention [21]. For a given target subject, achieving similar accuracy for that subject using a BCI trained using other subjects’ data, as shown in Fig. 3, compared to a BCI trained using the target subject’s data would imply that the trained BCI can be used without the need for any training data from the target subject. This could significantly simplify the setup of BCI systems and enhance their usability and scalability.

GAI techniques have been explored in the task of generating subject-independent data that could be used to train machine learning classifiers used in BCIs. GAI-based approaches were used to enhance P300-based BCI cross-subject performance, where hybrid CNN-LSTM GAN, DCGAN, cDCAGN and WGAN-GP GAN were examined in this task by balancing the dataset overcoming the imbalance between P300 and non-P300 samples [31, 33, 62]. The hybrid CNN-LSTM GAN showed the best performance in this case increasing the cross-subject accuracy by 10%. A similar idea was explored to enhance SSVEP-based BCIs in terms of their cross-subject performance. DCGAN, WGAN, ACGAN and VAEs were examined in this task resulting in performance improvements in the range of 3–35% [36, 63]. Finally, in MI-based BCIs, transformer models were examined with attention mechanisms enhancing the cross-subject performance in the range of 0.88–2.11% [64]. A CNN-based GAN that uses common spatial pattern filtering was also examined in this task along with DCGAN and VAE; enhancing the overall cross-subject accuracy by 15.85% [65]. Further utilization of other GAI techniques could help in identifying subject-independent features that would eliminate the need for subject-dependent data recording.

### GAI for end-to-end BCI development

**Fig. 4:**
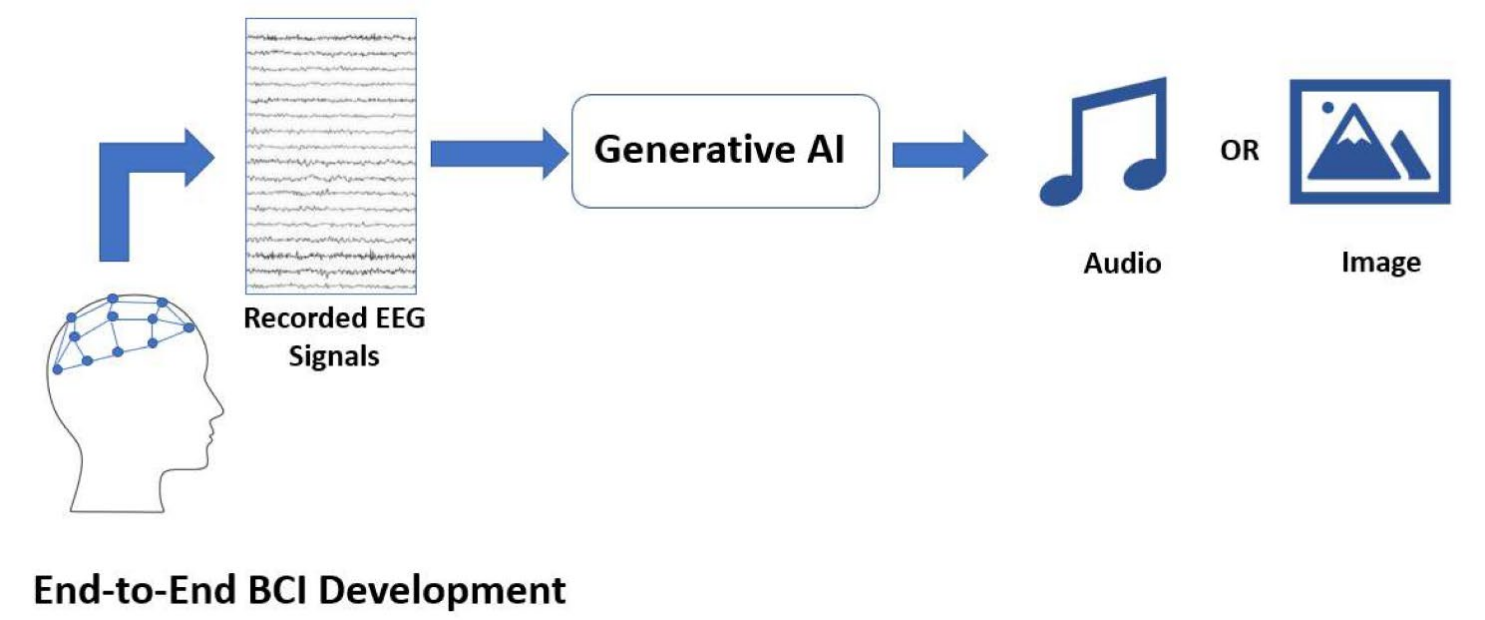
Using GAI for developing end-to-end BCI applications generating audio, images and video from EEG signals

The previous sections demonstrated how GAI techniques have enhanced already existing BCI paradigms that use brain patterns such as P300, SSVEP and MI. However, the advances in GAI could be leveraged to design end-to-end BCI applications as shown in Fig. 4, not only enhancing the training of the BCI systems or enhancing the resolution of the recorded EEG signals.

One end-to-end BCI application is generating speech from recorded EEG signals. This application could be of particular interest to subjects with reduced communication abilities. Generating speech from recorded EEG signals have been demonstrated to be an extremely challenging task given the complexity of human speech and the lack of clear understanding of the mechanisms that could map EEG signals to speech [66]. However, GAI techniques have been shown to provide a framework that could succeed in this task. To achieve the objective of generating speech from recorded EEG, a number of studies examined first using GAI to generate heard speech from recorded EEG. A dual GAN architecture was used in this task achieving a similarity of 78.5% between the actual and generated speech for both single words and long sentences [67]. A hybrid CNN-RNN along with HiFi-GAN was examined in generating spoken as well as imagined speech from EEG signals demonstrating a strong similarity between the spectrograms of both with a root mean square error of 0.19 [68]. Denoising diffusion probabilistic models (DDPMs) were also used exceeding the accuracy of other models in generating speech by 14.5% [69].

The extreme success GAI techniques demonstrated in generating realistic images and videos has inspired multiple research groups to explore the use of GAI techniques to generate images from EEG signals. Pre-trained stable diffusion models, VAEs and GANs were demonstrated to generate images from EEG signals [70, 71]. Stable diffusion models were shown to outperform other GAI techniques achieving a similarity of 45.8% between the actual and generated images. Another diffusion-based model that extracts multi-level semantics from recorded EEG signals achieved an accuracy of 85.6% when the generated images were evaluated using semantic classifiers [72]. A dual autoencoder was also employed in this task that is based on a CNN architecture [73]. Using semantic classifiers, images generated using this approach were classified with an accuracy of 85% using both inter-subject and intra-subject data. Another study demonstrated the use of ACGAN with attention modules in the same task reaching an accuracy enhancement of 22% compared to using an ACGAN architecture without attention [74].

## Conclusions and future perspectives

While BCI has traditionally been considered a technology that allows people with motor disabilities to interact with computers, it is now considered as one emerging human-computer interaction (HCI) technology that could be used in the future by all users. Adopting BCI as a novel HCI technology for general users is expected to attract more research efforts in this domain, which will have a positive impact on the development of BCI application developed as assistive technologies for people with disabilities. A glimpse of the increased attention BCI is now given is manifested in the recent utilization of GAI techniques in this domain attempting to overcome challenges that hinder the smooth operation of current BCIs. In this article, four of these challenges were discussed; namely, limited data availability, low signal resolution, inter-subject variability and developing end-to-end BCI applications. Examples of GAI techniques that were recently utilized to tackle these challenges are reviewed in this article. In all four challenges, GAI techniques resulted in a significant enhancement in the performance of different BCI applications, which opens the door to further development of GAI techniques in this domain.

While GAI has been used, as demonstrated in this article, to augment recorded EEG data used in training machine learning classifiers in various applications, GAI techniques could also be explored in augmenting the test data. For instance, in P300 speller applications, the flashing of each row and column of characters is typically repeated for 10 to 15 times, and the corresponding recorded training signals are averaged across flashings (trials). This is necessary to overcome the low signal-to-noise ratio encountered in single trials. This process is also performed during the online use of the speller system, which requires significant amount of time in the order of minutes to type a single word [75, 76]. One venue in which GAI techniques could be utilized is to generate additional trials from a few recorded trials during the online use of the system. For instance, signals corresponding to only 5 trials could be recorded and then use GAI techniques to generate additional trials to be averaged with the signals of the recorded trials. This could reduce the time needed to type using P300 speller systems and hence enhance the information bit rate and the overall user experience. Another direction that could be pursued, in addition to speech and image synthesis discussed in this article, is video synthesis from EEG signals. The success different GAI techniques demonstrated in image synthesis from EEG signals indicates that video synthesis could be feasible. This can be further utilized in futuristic end-to-end BCI applications that aim at generating subject-specific creative content such as art or music.

The success of GAI techniques in enhancing BCI applications as shown here encourages further adoption of GAI to overcome other challenges associated with BCIs. One venue where GAI can be fused in the development of BCIs is to use Large Language Models (LLMs) to enhance the performance of BCIs used as spellers. LLMs have recently been demonstrated to solve many long-standing problems in natural language understanding and processing [77]. LLMs could be utilized for speller BCIs by using LLMs to provide word or even sentence suggestions based on a few EEG-based character selections made by a BCI user. While the use of language models in character and word predictions has been introduced before to enhance BCIs [78], the use of LLMs with BCIs is yet to be fully explored.

Despite the promise GAI holds in developing BCIs, a number of challenges need to be alleviated in the near future to leverage the full capabilities of GAI. First, GAI requires extensive training data to obtain reliable and realistic synthetic outputs [79]. While one of the main applications of GAI techniques is to augment limited data, training GAI models with limited data could result in overfitting which limits the ability of GAI techniques to generalize. For subject-dependent BCI applications, recording extensive data from each subject is challenging since it requires long-duration recording sessions during which subjects would typically have limited ability to move to avoid EEG motion artifacts [80]. However, recording such data might be feasible given the advances in consumer-grade EEG devices which target developing portable EEG recording headsets for daily use. This could enable GAI techniques to achieve further improvement in BCI applications performance. Additionally, applying GAI techniques to enhance the cross-subject performance of BCI applications requires the availability of a dataset with a large number of subjects. This dataset needs to be of sufficient size to provide GAI techniques with enough data to be used to extract subject-independent generalized representative features. Currently, most datasets used are in the order of tens of subjects. A large-scale study that collects data from hundreds (or ideally thousands) of subjects could be the key to training GAI techniques that overcome inter-subject variability, hence allowing BCIs to get closer to widespread adoption as a reliable assistive technology. Second, the convergence and stability of GAI techniques training cannot be easily achieved, which typically requires extensive hyperparameter tuning [81]. This might not be feasible for BCI applications since it would require performing hyperparameter tuning for each subject which requires significant computational resources. This would further complicate the calibration phase of BCIs. Additionally, using GAI techniques to generate data for BCI applications in real-time is another related challenge since GAI techniques are characterized by their inherent computational complexity. Third, GAI techniques have been demonstrated to hallucinate if their function is not controlled [82]. While this might not result in severe consequences in other domains, GAI hallucinations could represent a major problem in the context of BCI development since BCIs could be used in critical applications such as controlling the movement of a wheelchair or a robotic arm. A hallucinating GAI could lead to a life-threatening situation in this case. Finally, GAI techniques used in BCI development, as demonstrated in this article, are predominantly GAN-based. The utilization of other GAI techniques is yet to be explored. Tackling the aforementioned challenges could pave the way for further utilization of GAI techniques in BCI development, which could transform the field leading to the wide-spread use of BCIs.

Finally, while GAI can contribute to revolutionizing BCI development as demonstrated in this article, BCI could also play a role in developing GAI techniques. For decades, the brain has represented an inspiration to the development of AI models leading to significant strides in the evolution of AI models [83]. However, there is a gap between how AI models are trained and operate compared to how the brain learns and processes information [84, 85]. This gap is argued to be one of the reasons AI is still not capable of reaching the general cognitive abilities of humans. BCI could bridge this gap by recording human brain activity in different tasks which can be then used to train GAI techniques to generate embeddings that resemble the brain representation of information. Such a brain-in-the-loop approach could provide GAI techniques with additional dimensions of information representation, which could augment the capabilities of GAI techniques. Thus, the benefits of integrating GAI and BCI are bi-directional that can lead to significant enhancements in both domains in the near future.

## Data Availability

Not applicable.
